# "Ierne" on Guard

**Published:** 1919-11-29

**Authors:** 


### "IERNE" ON GUARD.

It is hard luck for an editor to have to explain, or rather to explain away, the words of his correspondent. Mine appear to have created a little storm, which I do not altogether regret, strange as it may appear. I prefer the storm to the mist, and, as they are hostile elements, they cannot exist side by side.

What I said may be explained, but not explained away. I was not "obsessed by anger." My words were deliberate, and the "Zealous College Supporter" who describes them as "absolutely untrue" quoted and interpreted me correctly. Referring to the many societies to which the nurse had subscribed, and which, according to my description, have so far failed to minister to her "pressing necessities," I included the College!

Open confession, they say, is good for the soul. So now you have my unreserved plea of "Guilty."

I particularly desire to explain myself, and in so doing I wish to claim a front place among "zealous College supporters." With clear conscience I can say that I doubt if the College has had any more ardent supporter than I, a statement which can be verified by reference to The Hospital back numbers. Moreover, anything that I have in the past stated about the College I still maintain. In its design and conception it is Utopian. But no human institution is perfect, and no community of people infallible. Hence I hold that the best friend that the College can possess is the critic who has the honesty to point out potential weaknesses, errors, or defects. Criticism I hold to be the finest and most wholesome medicine that public men and women can use. If the critic is prompted by motives that are unworthy, so much the worse for the critic. If he is honest, be he right or wrong, he deserves a hearing. I claim to be heard.

### Golf and Nursing.

It may seem a long way from nurses and nursing, but may I ask the reader—Have you ever played the game of golf on a strange links accompanied by an intelligent caddie? Two things occupy your thoughts—first, that you have an opponent, and, secondly, that you want to get your ball on the green. You are always, or almost always, in a dilemma. With the best possible of intentions the most skilful player is often in worse plight—not only in a dilemma, but also in a bunker. In difficult quarters a wise caddie is a great help. It may be a fairly long shot, and you are not quite sure about the distance. You take your mashie and you look at the caddie with a note of interrogation in your eye. "Take a brassie," he advises, "and let out." The player who is a sport never gets cross with his caddie if he knows him to be a loyal servant. He may not follow his advice, but he gives it consideration. He knows it to be well meant, and he realises that it may be sound. Now, Mr. Editor, and readers all, *I am that caddie*! I am not the College; I am not The Hospital. I am in the game, however, and all that I ask for is a fair hearing. I note that Sir Arthur Stanley describes this as a crisis in the nursing profession, He is right. It is a crisis. It is also a crisis in the history of the College. I hold the view that the College is trying to play a brassie shot with a mashie. If any mistake is made the opponent may win the hole, and may possibly also win the match.

### The Nurses' "Pressing Necessities."

I have read with admiration Sir Arthur Stanley's dignified letter to College members, in which he catalogues achievements of which any institution might be proud. Sir Arthur is a master builder, and if he errs when he speaks it is always on the side of modesty. By his zeal and influence he has wrought a miracle for the nurse. He is the first person since the days of Florence Nightingale to make a great and successful effort to lift her out of the miry clay, and to set her feet upon a rock. The College, its inquiry bureau, its scheme of higher professional education, its Endowment Fund, the Nation's Tribute, and, above all, the national and imperial ideal of the College, is all Sir Arthur Stanley's creation, and who shall dare to belittle it? Far be the thought that I should find myself associated with "noisy opposition and unscrupulous attacks."

Nevertheless, none of these, I venture to observe, are the "pressing necessities" of the nurse. The nurse that I am thinking of is not the nurse in distress, not the aged, disabled, or infirm, not the matron even, but the woman in the wards. Her "pressing necessities" are threefold—(1) better pay; (2) shorter hours; (3) a career in prospect. To-day, here, and now, the nurse in the wards makes a claim for these things. I hold that she is justified. Measure her hours of toil, her responsibilities, her outlook, her wages, by any British standard, and it will be immediately obvious that she is outclassed by every other group of women workers. The College authorities know it. They have prepared a first-class document on the pay question, after careful investigation. But no effort has been made, so far as I am aware, to insist on the scale being recognised. The question of hours of toil, and that of a career, remain untouched. The invitation of the Minister of Health has not been answered. That there are difficulties in the way I am aware. I am not blind. It is the immediate duty of the College to face the difficulties and to surmount them. If they are not surmounted the standard of probationers will fall, and nurses in harness will join the trade union. I am not a prophet, but a witness of what is now happening.

### The Weak Spot.

The employers of nurses are Hospital Committees. The managing directors of the College are hospital matrons. Herein is the crux of the whole matter. I have the utmost sympathy for the matron. She may have a heart of gold, but she is utterly powerless. I have experience of Hospital Committees, and I know. The "pressing necessities" of nurses do not come before Hospital Committees unless they are thrust upon them. Articles in The Hospital do not appear on the agenda. A matron dare not, at the risk of her office, appear at committee meetings as the voluntary champion of her subordinates. There may be, and doubtless are, rare exceptions. Now the College authorities apparently assume that their model salary scale will influence Hospital Committees. This is what I call playing a brassie shot with a mashie. I ought rather to have said with a putter. This salary scale is not discussed at Hospital Committees, and will be absolutely ineffective if not followed up with pressure. The College people must "let out." That Hospital Committees are faced with difficulties in no way affects the issue. Hospital Committees have no right to rob the British nurse of her earnings, and it is the duty of the College to champion her cause. Fifteen thousand nurses are the bricks that compose its walls. I believe, and have always believed, that the British public will pay the nurse if asked to do so.

I do not know whether or not the College has hesitated to bring the necessary pressure to bear in the proper quarter concerning salaries and hours of employment, because its Executive are hospital matrons. But this I do know—that the nurse expects the Executive at least to take her into their confidence and explain the reason.

IERNE.

